# The Effect of Simvastatin on Lowering Panel Reactive Antibody Titer in Sensitized Dialysis Patients: A Randomized Placebo Controlled Clinical Trial

**Published:** 2010-05-01

**Authors:** J. Roozbeh, A. Sattarinezhad, R. Afshariani, A. Eshraghian, M. M. Sagheb, G. Raeesjalali, S. Behzadi, S. Nikeghbalian, M. Salehipour, H. Salahi, A. Bahador, S. A. Malek-Hosseini

**Affiliations:** 1 *Organ Transplant Research Center, Nemazee Hospital, Shiraz University of Medical Science, Shiraz, Iran.*; 2 *Department of Internal Medicine, Nephrology Urology Research Center, Shiraz University of Medical Sciences, Shiraz, Iran. *; 3 *Department of Health and Nutrition, School of Health, Shiraz University of Medical Sciences.*

**Keywords:** Panel reactive antibody (PRA), Renal transplantation, Simvastatin, Dialysis, End-stage renal disease

## Abstract

Background: Patients with panel reactive antibodies (PRA) have many difficulties to find a crossmatch-negative kidney for transplantation and are at a higher risk of post-transplantation rejection.

Objective: To evaluate the effect of simvastatin on PRA and post-transplant outcome of these sensitized patients.

Methods: 82 patients with end-stage renal disease (ESRD) with a PRA ≥25% were evaluated. In a one-year follow-up, the patients were treated with simvastatin. These patients were compared with 82 matched controls receiving placebo tablets. At the end of the second and 12^th^ month, PRA was rechecked in all patients. Those patients who underwent transplantation continued to take simvastatin six months after transplantation. Serum creatinine levels were checked at monthly intervals post-operation.

Results: The mean±SD PRA level at the end of the second month was 36.63%±31.14% and 45.34%±24.36% in cases and controls, respectively (P=0.012). Seven patients in the case group and 10 in the control group were lost to follow-up. The remaining patients continued to take simvastatin for 12 month.

The mean±SD PRA level at the end of the 12^th^ month was 24.02%±31.04% in cases and 43.15%±26.56% in controls (P=0.001). 25 patients underwent renal transplantation and continued to receive simvastatin 6 months after transplantation. These patients were matched with 25 controls treating with placebo. The mean±SD creatinine level 6 months after kidney transplantation was 2.05±1.14 mg/dL and 3.15±1.09 mg/dL in cases and controls consecutively (P=0.02).

Conclusion: Simvastatin can be safely used to lower PRA and improve post-transplantation outcomes.

## INTRODUCTION

It has been proved that presence of panel reactive antibodies (PRAs) in the sera of renal transplant candidates is associated with hyperacute or delayed humoral immune responses against the graft after transplantation [1]. In addition, these sensitized patients must wait for a long time to find a crossmatched negative kidney as donor for transplantation [2]. As a result, some modalities such as plasmaphresis and intravenous immuonoglobulin (IVIG) in combination with immunosuppressive drugs have been used to overcome this problem [3]. Recently, use of statins—antihyperlipidemic agents—like simvastatin, pravastatin, *etc.* has been proposed to be a safer and more effective method for desensitization [4-6]. This study was conducted to investigate the effect of simvastatin, an HMG-CoA reductase inhibitor, on lowering serum PRAs in highly sensitized renal transplant candidates and to evaluate its role in prevention of acute rejection after renal transplantation.

## MATERIALS AND METHODS

Patients:

From 750 patients with end-stage renal disease (ESRD) who were in the waiting list for renal transplantation or referred to our nephrology clinic in Shiraz Transplant Center, 164 patients who met our criteria, were included in this study. Using a simple random method, these patients were randomized to the patient and control groups, each consisted of 82 patients.

Inclusion criteria:

- Patients from both sexes aged from 18 to 75 years old.
- Patients with ESRD on hemo- or peritoneal dialysis. And,
- PRAs ≥25%.

Exclusion criteria:

Pregnant women, patients who need ongoing blood products, patients taking other treatments to decrease PRA, patients listed for multi-organ transplant other than kidney as well as those with liver failure were excluded from the study.

Lab tests: 

Baseline aspartate aminotransferase (AST) and alanine aminotransferase (ALT) were checked for all patients in the case group. Then, all of the 82 patients received 20 mg/day oral simvastatin for two months. Patients in the control group received the same dose of the same shape starch tablets as placebo during these two months. At the end of the second month, PRA was checked for all the patients again. PRA testing was done using the complement dependent cytotoxicity (CDC).

We continued treatment with simvastatin for 12 months by checking liver enzymes (AST, ALT) every two months and PRA at the end of the 12^th^ month. In patients who complained of myalgia and muscle weakness, creatine phosphokinase (CPK) was also determined to watch any side effects of simvastatin. The control group were also treated with placebo tablets for 12 months. The complete response to simvastatin was defined as a drop of PRA to <25%. Rejection episodes were proven by kidney biopsy.

Statistical analysis:

All parameters were expressed as mean±SD and analyzed with SPSS software for Windows version 12.0 (SPSS Inc, Chicago, IL) using *Student’s t* test for paired data and χ^2^ test for comparison of categorical data. A P value <0.05 was considered statistically significant.

Ethics and consents: 

The study protocol was carried out in accordance with the Helsinki Declaration revised in 1989. All subjects were informed about the study protocol and written consents were obtained from all participants. Ethics committee of Shiraz University of Medical Sciences approved the study.

## RESULTS

There were 82 cases and 82 controls. The mean±SD age of patients was 39.3±11.3 years in cases and 37.8±12.1 years in controls (P>0.05). There were 50 (61%) men and 32 (39%) women in case group and 47 (57%) men and 35 (43%) women in control group (P>0.05). Seventy-eight (95%) patients in case group and 78 in control group were on hemodialysis program for a mean±SD duration of 26.56±18.28 months three times a week. Four patients in case group and four in control group were on peritoneal dialysis program for a mean±SD duration of 195±21.48 months. In each group, 32 (39%) patients had history of previous renal transplantation and 11 had history of previous transfusion of blood products. The causes of ESRD are outlined in Table 1.

**Table 1:** Causes of end-stage renal disease in the case and control groups

Cause	Cases	Controls
Acute tubular necrosis Leading to ESRD^a^	5	4
Renal artery stenosis	2	2
ADPCKD^b^	2	3
Chronic tubulointrestitial nephritis	2	2
Nephrolithiasis	4	3
Diabetes mellitus	8	9
Malignant hypertension	8	8
Lupus nephritis	10	8
Glomerulonephritides other than diabetes and Lupus	14	14
CRF^c^ with unknown etiology	27	29

a End-stage renal disease**,**

b Autosomal dominant polycystic kidney,

c Chronic renal failure

**Figure 1:**
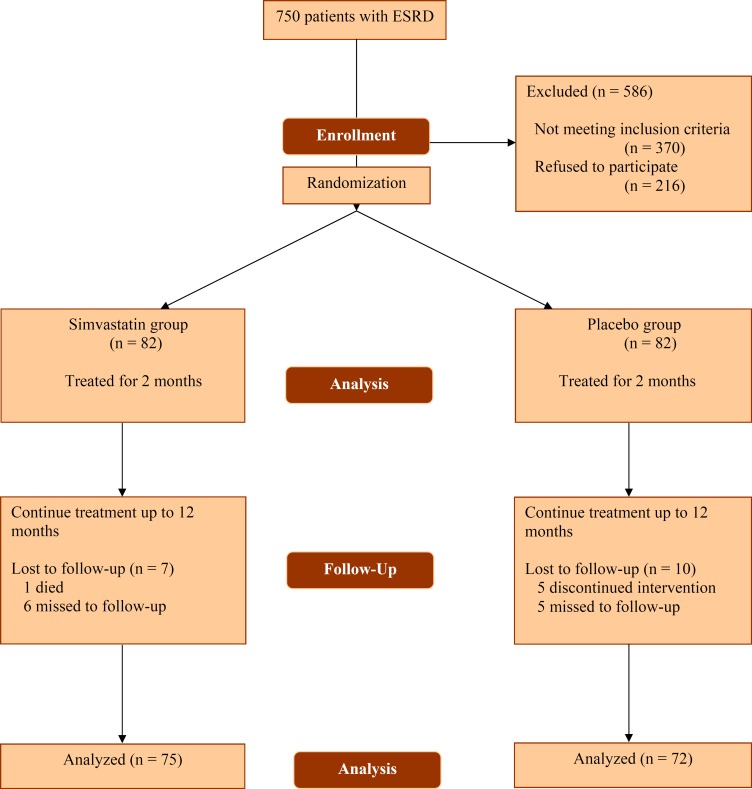
Flow diagram of the study

All of the 82 patients in case group received simvastatin for two months. Eighty-two controls received placebo tablets during this period. The mean±SD PRA level at the end of the second month was 36.63%±31.14% and 45.34%±24.36% in cases and controls, respectively. The mean PRA level decreased significantly (P=0.012) after two months of treatment with simvastatin. The mean±SD drop was 14.22%±19.63% (range: 9.90%–18.53%); in the case group, 28 (34%) patients showed a complete response to the treatment (*i.e.*, PRA<25%). We continued simvastatin treatment up to 12 months. During this period, six patients in the case group and 10 in the control group discontinued the follow-up program; one case deceased (Fig. 1).

The mean±SD PRA level at the end of the 12^th^ month was 24.02%±31.04% in the case group and 43.15%±26.56% in controls (P=0.001). The mean±SD drop in PRA in cases was 26.57%±26.10% at the end of 12^th^ month (Table 2).

**Table 2:** Mean±SD PRA level before and 2 and 12 months after simvastatin therapy

Time	Mean PRA^a^ level (%) in		P-value
	Cases	Controls	
Before treatment	50.85±24.14	48.36± 36.71	NS*
2 months after treatment	36.63±31.14	45.34±24.36	0.012
12 months after treatment	24.02±31.04	43.15±26.56	0.001

a Panel reactive antibody,

* Not significa

Overall, 53 patients showed complete response to the treatment compared to 12 controls (P=0.03). Twenty-five (33%) of 75 patients underwent renal transplantation after a mean±SD period of 6.45±3.56 months since beginning of simvastatin therapy. Eleven patients received their kidneys from living unrelated donors, five from living related and nine from deceased donor in both cases and controls. These 25 patients were matched with 25 renal transplanted controls. From these 25 patients, three were lost to follow-up. Other 22 transplanted patients were followed for six months after transplantation receiving the same dose of simvastatin. The controls received placebo for six months and all of them completed the study. The mean±SD serum creatinine level six months after kidney transplantation was 2.05±1.14 and 3.15±1.09 mg/dL in cases and controls, respectively (P=0.02). The mean creatinine level at the end of the study (1.37±1.61 mg/dL) showed a significant reduction in comparison to the initial values (P=0.001). 

Two patients presented with graft loss and became dependent on hemodialysis again. Others remained dialysis free with well functioning kidneys. None of the patients presented with liver failure or rhabdomyolysis during the follow-up period. 

## DISCUSSION

At the first stage of this study, we treated sensitized ESRD patients with simvastatin. Then, we continued simvastatin therapy in those who underwent kidney transplantation. Glomerulonephritis and lupus nephritis comprised a significant proportion of known causes of ESRD in sensitized patients. This study revealed that simvastatinn treatment in cases decreased PRA level significantly compared to the controls treated with placebo. Simvastatin also improved the outcomes after kidney transplantation.

Interesting results have been reported in clinical trials investigating immunomodulatory role of simvastatin. Truncer, *et al.*, reported that the rate of acute rejection was lower in renal transplant patients using simvastatin and pravastatin in comparison with those patients who did not receive these medications [7]. Other studies suggest that pravastatin also decreases the incidence of both clinically severe acute rejection episodes and natural killer cell cytotoxicity after orthotopic heart transplantation [8, 9]. Results of another study by Ozdemir and co-workers revealed that continuous simvastatin therapy is effective for treatment of highly sensitized patients and is also effective on graft survival of this group of patients [4, 5]. Despite investigations in favor of the role of HMG-CoA reductase inhibitors in treatment of sensitized patients, it is still a topic of controversy. Ossareh, *et al*., did not observe any useful effects with lovastatin [10]. Mahmoud, *et al*., reported that administration of neither IVIG nor simvastatin alone cannot effectively inhibit preformed HLA-antibodies to allow successful renal transplantation [11].

This study provided evidence suggesting the role of simvastatin in decreasing PRA level. It also showed that simvastatin may improve the outcome of patients after renal transplantation. Larger sample size and proper duration of follow-up are strength of our study compared to other similar studies conducted on this issue. Considering the presence of many confounding variables including age of patients, causes of ESRD, causes of sensitization, type and duration of dialysis as well as limited number of patients in each categories, there are few controlled trials regarding the role of HMG-CoA reductase inhibitors in the treatment of sensitized patients.

Further studies, especially large placebo controlled trials may be more useful and conclusive regarding the use of simvastatin in the treatment of sensitized patients before transplantation along with its usage as a protective medication in acute rejection after renal transplantation.
